# Associations of subjective age trajectories with loneliness and stress across adulthood

**DOI:** 10.1371/journal.pone.0320673

**Published:** 2025-04-01

**Authors:** Anna E. Kornadt, Markus Wettstein, Anthony Lepinteur, Claus Vögele, Conchita D’Ambrosio

**Affiliations:** 1 Department of Behavioral and Cognitive Sciences, University of Luxembourg, Esch-sur-Alzette, Luxembourg; 2 Department of Psychology, Humboldt University Berlin, Berlin, Germany; Universiti Putra Malaysia, Malaysia

## Abstract

Subjective age, that is the age a person feels like in relation to their chronological age, is indicative of a variety of biological, psychological, and social aging processes. Despite its importance, studies that investigate multi-variate, dynamic, longitudinal relations of subjective age with its potential determinants and potential mechanisms of these relations have so far rarely been employed. In the current study, we focus on loneliness as a potential subjective age determinant. As loneliness affects a variety of psychosocial and health outcomes across life and is stereotypically perceived as a feature of old age, we investigate whether loneliness is related with levels and changes in subjective age. We furthermore test whether this association is mediated via self-reported stress. *N* =  5,594 participants aged 18 – 93 years (*M*_age_ =  50.41, *SD* =  15.99) who participated in a longitudinal survey comprising up to three measurement occasions over a time span of 2.5 years reported their loneliness, subjective age, and stress as well as sociodemographic and health-related covariates. We employed latent growth modeling and found that, when controlling for sociodemographic and health-related covariates, higher loneliness was related to an older subjective age cross-sectionally and to a steeper increase in subjective age over time. These relations were mediated via stress; however, the relation between stress and subjective age was no longer statistically significant when including the covariates. All associations were qualified by significant interactions with chronological age, albeit in different directions. Our findings attest to associations between loneliness, stress and subjective aging experiences and highlight the need for an age-informed approach when planning further studies and interventions.

## Introduction

As people move through the lifespan, their chronological age, and the age they feel like are by no means identical. How old someone feels is termed subjective age [1] and starting in their late twenties, up to very old age, most people feel younger than their chronological age (e.g., [2]). Feeling younger is usually adaptive for later life outcomes and has a positive impact on health, cognitive functioning, and longevity [3,4], for instance via behavioral (e.g., physical activity; [5]) and physiological (e.g., inflammation; [6,7]) pathways. Thus, subjective age is an important predictor of developmental trajectories.

Given its high predictive power, the question of what affects subjective age has guided much research in recent years. According to theoretical elaborations and empirical findings, subjective age seems to reflect a variety of biological, psychological, and social processes and it is thus also termed a “biopsychosocial marker of aging” [8]. Nevertheless, even though several predictors have been identified, studies that investigate multi-variate, dynamic, longitudinal relations and processes of potential subjective age determinants have thus far rarely been employed. Two factors that might be highly relevant predictors of subjective age are loneliness, defined as the subjective sense of inadequate social relations (e.g., [9,10]) and stress (both self-reported and measured via physiological indicators; [11,12]. Given that loneliness has been associated with increased stress levels (e.g., [13,14]), one could argue that this is one mechanism underlying the effects of loneliness on subjective age. This may have been especially pertinent in the context of the Covid-19 pandemic, when both temporarily elevated stress and loneliness levels were observed in certain populations (e.g., [15–20]). Subjective age also fluctuated with pandemic progression, with typically younger subjective ages at times of higher COVID-19 infection rates [21–25].

In the current study, we investigate whether loneliness is associated with subjective age trajectories via perceived stress and whether this is true cross-sectionally as well as longitudinally, i.e., for levels as well as changes in stress and subjective age. Moreover, given the differential relevance and impact of both stress and social relations across life, which are described in established theoretical frameworks such as Socioemotional Selectivity Theory [26] or Strength and Vulnerability Integration [27], we analyze whether the associations of loneliness, stress and subjective age vary according to chronological age. We examine these research questions with data of the COME-HERE study, which followed a large group of survey participants in a broad age range (18 to 93 years) from five countries over the course of the Covid-19 pandemic and beyond.

### Loneliness as a developmental risk factor throughout adulthood

Loneliness has emerged as a focal psychosocial variable of interest in the past years, and researchers as well as policymakers have addressed the “loneliness epidemic” [28] as a public health issue [29]. Studies on the prevalence of loneliness across the life span find that problematic levels of loneliness can be observed in many countries and across all age groups [30,31]. While many studies find that loneliness seems to be especially pronounced in late adolescence and very old adulthood ([32,33]), a recent meta-analysis of longitudinal studies found mean-level stability of loneliness across adulthood [34]. Furthermore, the prevalence of loneliness in midlife seems to increase historically, however, not uniformly for all countries [35].

The experience of loneliness has detrimental consequences for health, well-being, and development. People experiencing loneliness are at higher risk for depression, physical health problems and functional limitations [36,37]. One pathway through which loneliness can impact health and well-being is through its link with self-reported and physiological indicators of stress ( [13,36,38–41]). In general, higher loneliness has been shown to increase cardiovascular reactivity (e.g., [13]); however, evidence on this relationship is somewhat mixed ([42]). At the molecular level, loneliness is associated with altered stress hormone signaling and increased inflammation [43].

### Loneliness and subjective age

Given that subjective age seems to be a marker for different biological, social, and psychological aging processes, there are several lines of argumentation that support the assumption that loneliness might predict subjective age. Loneliness is per definition an unwanted state, signaling that social relations are not corresponding to the current needs of a person ([44]). Thus, people who are lonelier might have less social support and social resources to tackle age-related changes and challenges, which might result in more negative developmental trajectories and, therefore, older subjective ages [9]. Furthermore, given the detrimental effect of loneliness on health, and the fact that health problems are related with older subjective ages ([1],[45]), health might be a mechanism via which loneliness affects people’s subjective age. Relatedly, a study by Lynch and colleagues [46] found that loneliness was related to accelerated biological aging, as indexed by DNA methylation (see also [47]). This physiological mechanism might also be implemented in older subjective age. Loneliness is among the most pervasive negative stereotypes of old age, and many people are concerned with becoming lonely as they grow older [48,49]. Thus, the experience of loneliness might be seen as the fulfilment of these stereotypic fears and thus increase subjective age, as individuals might interpret loneliness as a side effect or consequence of “being old” and in consequence feel older once they are affected by loneliness.

Accordingly, several studies have empirically addressed the relationship of loneliness and subjective age. Cross-sectionally, loneliness is consistently related to older subjective age (e.g., [25,50–52]). There are fewer longitudinal studies investigating directional relations and changes over time, with somewhat diverging results. In a study by Kleinspehn-Ammerlahn and colleagues [10] with very old participants from the Berlin Aging Study, surprisingly, higher loneliness was related to a younger subjective age, however, they controlled for various cognitive and health-related variables which are potentially related to both loneliness and subjective age. In contrast, Ayalon and colleagues [9] used data from the Health and Retirement Study and found that a decrease in loneliness was related to an accelerated decrease in subjective age over four years. That is, participants whose loneliness decreased, had a more pronounced reduction in their perceived age over time. Overall, evidence points to an association of greater loneliness with an older subjective age; however, more longitudinal evidence is needed. Particularly, pathways through which loneliness might impact subjective age are not well understood, and little is known regarding the role of chronological age for loneliness-subjective age associations.

### Stress as a potential link between loneliness and subjective age

Loneliness can impact health and developmental outcomes via several pathways, such as behavior, cognition, or altered brain activation in response to social stimuli [53]. Another important mechanism is the impact of loneliness on neurobiological mechanisms, such as stress. Stress is also highly relevant for subjective age and might thus be one potential factor that links loneliness to subjective age. Loneliness has been shown to increase stress (e.g., [13,14,43]), especially for older adults [53] and there is also a large body of evidence showing that stress might lead to accelerated biological aging [54]. Stress has also been associated with older subjective ages [55,56], with evidence for the predictive effect of stress on subjective age from both long-term longitudinal and micro-longitudinal studies [57]. For instance, Wettstein and colleagues [12] found that in a sample of adults aged 40 – 85 years, higher perceived stress was related to an older subjective age three years later. This was especially pronounced for chronologically younger compared to chronologically older adults, which implies that stress-subjective age associations might vary according to chronological age. In a study by Terracciano and colleagues [21] which was conducted over the course of the Covid-19 pandemic, more stress was related to an older subjective age; however, stress did not predict change in subjective age. Further evidence shows that stress is related to and predicts subjective age in daily and momentary contexts [57]. On days and in moments when people feel more stressed, they also feel older, and higher momentary stress predicts higher subjective age at the next measurement ([1,11]). Thus, given the substantial associations between loneliness, stress and subjective age as identified in prior research, it is conceivable that loneliness is related with levels and trajectories of subjective age via stress.

### The role of chronological age

Prominent theoretical frameworks have addressed the differential importance and impact of both social relationships and stress across the lifespan, making it highly relevant to take chronological age into consideration when addressing the current research questions. Socioemotional Selectivity Theory [58] for instance postulates that goals and motivation are closely linked to decreasing time perspective, with reduced time perspective leading to a priorization of emotion regulation and close social relationships. Advancing age, which comes with reduced time perspective, thus means that close social relationships become more important for well-being. This phenomenon has implications for the impact of loneliness on health-related outcomes and stress across the lifespan. For instance, findings show that while loneliness seems to have detrimental implications throughout life (e.g., [37]), some consequences might be especially pronounced in later life. Even though older adults per se are not lonelier than adults from other age groups, they are, once loneliness has set in, at higher risk for chronic loneliness [44,59] and might also experience more severe health consequences associated with loneliness [42,53].

Relatedly, in terms of stress, the model of strength and vulnerability integration (SAVI; [60]) postulates that while older adults might be better in employing strategies to avoid or mitigate stressors, those stressors that accumulate in later life and that cannot be avoided have more detrimental consequences [61,62]). The loss of social relationships due to age-related changes and circumstances, which might not be under the control of the individual might represent such a stressor [27]. Furthermore, SAVI postulates that due to reduced physiological flexibility, chronic stress, induced by factors such as lasting loneliness, is more difficult to regulate for older compared to younger adults [60], which might exacerbate the negative effects of loneliness and stress on psychological and health-related outcomes described previously for older adults. Thus, one might expect a stronger effect of loneliness and stress on subjective age for older adults than for younger adults.

Predictions derived from SAVI and SST are not necessarily the same (see for instance [63]). Both SAVI and SST, however, also point out strengths of older adults in terms of their regulation of social networks and emotion regulation processes. Consequently, previous findings show that stress might have more severe implications for the subjective age of chronologically younger adults [12]. In addition, due to their advantages in strategies of coping and self-regulation to avoid stressors and enhance positive affect, older adults were for instance less affected by the pandemic in terms of stress and well-being ([26,63]). This might result in weaker associations between stress, loneliness and subjective age for older compared to younger persons.

### Aims and hypotheses

Taken together, the empirical evidence shows that higher loneliness and stress might be related to older subjective age and increases in subjective age over time. Still, there are only few longitudinal studies including participants in broad age ranges, and the interplay of all three variables cross-sectionally and over time has not been investigated so far. In the current study, we thus investigate the relationship of loneliness with levels and changes of stress and subjective age, as well as the indirect effect of loneliness on subjective age (levels and changes) via stress. By investigating the assumed relations for both levels and changes, we aimed to disentangle cross-sectional and longitudinal associations between variables, which have both been shown in previous research. Moreover, whereas cross-sectional associations might be influenced by situational context factors (e.g., a Covid-19 period effect, with a currently high COVID-19 infection rate triggering both stress due to fear of infection and loneliness due to contact restrictions), longitudinal associations are less dependent on such context factors, and they describe to what extent changes across a longer period of time are interrelated. Finally, more compelling evidence regarding mediation effects can be derived if longitudinal, rather than cross-sectional, associations are considered.

Note that our operational definition of stress focuses on self-reported stress, that is to what extent individuals feel stress [64]. Such a stress measure does not necessarily correspond to biological stress indicators such as cortisol levels or physiological stress reactions ([65]); however, stress appraisals are highly relevant for the coping strategies that are chosen [66], and they have been found to be meaningfully related with subjective age ([11,12]).

We will base our analyses on a study that assessed loneliness, stress, and subjective age over a time span of 2.5 years, in the context of the Covid-19 pandemic in an age-heterogeneous sample comprising adults aged 18 to 93 years. During the pandemic, with its fluctuating requirements of social distancing, the absolute disruption of routines and high insecurity [67], as well as the portrayal of older adults as a risk group for severe progression of the Covid-19 disease [68], all three variables of interest might have been affected by a Covid-19 period effect, which makes them especially relevant to study.

We hypothesize that:

1. Higher levels of loneliness are related to feeling older and an increase in subjective age over time.
2. Higher levels of stress and increases in stress over time are related to feeling older and an increase in subjective age over time.
3. There is an indirect effect of loneliness on subjective age (level and change) via stress (level and change).

While we will investigate the relationship between loneliness, stress, and subjective age across a broad age range in adulthood, we will also test the predictions from theoretical frameworks such as SAVI and SST by examining whether these relationships are affected by and moderated by chronological age. Given the mixed empirical findings of chronological age in terms of stress and loneliness as described previously, we do not derive concrete hypotheses for the direction of the age effects.

We included gender, education, depressive symptoms, physical health, and country of residence as covariates, considering their previously reported relations to all three variables of interest.

For reasons of transparency, we state that we initially planned to include loneliness also longitudinally. However, due to the high dropout at T11, covariance coverage was too low for statistical analyses with all three variables. Thus, we restricted our inclusion of loneliness to T4, and thus cannot investigate the impact of changes in loneliness on stress and subjective age. This did not change our general approach and other hypotheses.

## Method

### Sample and procedure

We used data from the COME-HERE longitudinal online survey [69]. COME-HERE started in April 2020 and ever since, participants are contacted every three months on average (more often in the first year) to report on their life, health, and well-being over the course of the pandemic and beyond. Currently, 12 waves of data are available spanning the time between April 2020 (wave 1) and March 2024 (wave 12). Participants (*N* =  8,063) were recruited via Qualtrics in France (*n* =  1706), Germany (*n* =  1720), Italy (*n* =  1710), Spain (*n* =  1711), and Sweden (*n* =  1216). Each country sample is nationally representative regarding age, gender, and region of residence. Respondents were aged 18 to 93 years at T1 (M_age_ =  47.46, SD =  16.97), 51.7% were female. More details on the sample and sample composition are presented in [69] and Table 1. Questionnaires were originally drawn up in English and translated by bilingual individuals into German, French, Italian, Spanish, and Swedish. COME HERE was approved by the Ethics Research Panel of the University of Luxembourg, decision number erp 20-026 c/a come-here.

**Table 1:** Descriptive statistics and bivariate correlations within and between timepoints for all study variables.

Variable (range)	Mean (SD)	1.	2.	3.	4.	5.	6.	7.	8.	9.	10.	11.	12.
1. Loneliness T4 (1-4)	2.03(0.66)	1											
2. Stress T4 (0-4)	1.52 (0.72)	**.65**	1										
3. Stress T8 (0-4)	1.47 (0.73)	**.55**	**.70**	1									
4. Stress T11 (0-4)	1.38 (0.73)	**.55**	**.66**	**.72**	1								
5. SA T4	-0.05 (0.27)	**.26**	**.26**	**.22**	**.24**	1							
6. SA T8	-0.04 (0.27)	**.25**	**.25**	**.28**	**.24**	**.51**	1						
7. SA T11	-0.03 (0.25)	**.26**	**.23**	**.26**	**.25**	**.54**	**.55**	1					
8. Age (18-93)	50.41 (15.99)	**-.28**	**-.34**	**-.34**	**-.31**	**-.35**	**-.36**	**-.30**	1				
9. PHQ T4 (0-3)	0.68 (0.69)	**.62**	**.67**	**.60**	**.60**	**.32**	**.32**	**.29**	**-.33**	1			
10. PHC T4 (1-8)	0.33 (0.70)	**.04**	**.03**	**.06**	.07	.02	.02	-.01	**.22**	**.10**	1		
11. Education (1-8)	4.92 (1.66)	-.01	-.00	-.01	-.01	.02	.03	-.06	**-.10**	.02	**-.04**	1	
12. Gender (% female)	50.3	.11	**.15**	**.14**	**.14**	**.05**	**.06**	.05	**-.16**	**.13**	**-.08**	**-.04**	1

Note: SA =  subjective age; PHQ =  depressive symptoms; PHC =  physical health problems; gender 0 =  male, 1 =  female; education 1 =  primary 8 =  PhD; bold coefficients are significant at *p* < .05. Age, gender, and education represent the N =  5,594 participants who were in the panel at T4.

Subjective age was measured at wave 4 in November 2020 (*N* =  5,594), wave 8, which took place 15 months later in February 2022, *N* =  3,644) and wave 11, another 16 months later in June 2023 (*N* =  740). Therefore, the present analyses are restricted to these three measurement occasions, and we used T4 as our baseline. As dropout was considerable at T11, we checked whether dropout at T11 was related to any of our study variables. Participants who stayed in the sample at T11 were older (*r* = .11, *p* < .001), had fewer depressive symptoms at T4 (*r* =  -.05, *p* < .001), were less lonely at T4 (*r* = -.03, *p* = .04) and reported less stress at T4 (*r* =  -.04, *p* = .05) and T8 (*r* =  -.06, *p* < .001), compared to those who dropped out. All significant effects were small [70].

### Measures

Participants were recruited from the Qualtrics on-line market research panel. They were contacted directly by Qualtrics and upon agreeing to participate, received the survey link. They answered several questionnaires on different topics, depending on measurement occasion. While at the beginning, pandemic-related topics such as working from home, or reaction to confinement measures were prominent, at later stages attitudes toward vaccination and continued teleworking were asked. Physical and mental health and distress, as well as a wide range of health behaviors were assessed at all time points.

#### Subjective age.

Subjective age was measured at waves 4, 8, and 11 with the standard single item: “Many people feel older or younger than their actual age. How old (in years) do you feel most of the time?”. Following standard practice (e.g., [71]), outliers of more than 3 standard deviations above and below the mean were removed (2% of all values). Due to the large age range of our sample, we divided the difference score (felt age – chronological age) by participants’ chronological age and used a proportional discrepancy score [8], which denotes the percentage of feeling younger or older. A proportional score of .20 thus indicates that a person feels 20 percent older than their chronological age, whereas a value of -.20 indicates that the person feels 20% younger.

#### Loneliness.

Loneliness at T4 was measured with the 8-item version of the UCLA loneliness scale [72]. Participants rated their loneliness in the past two weeks (e.g., “How often do you feel left out?”) on a four-point scale ranging from 1 “never” to 4 “often”. Two positively worded items were recoded, and a mean loneliness score was computed, with higher values indicating more loneliness. MacDonald’s Omega was high (Ω = .84).

#### Perceived stress.

Perceived stress was measured at all waves with the Perceived Stress Scale [64], one of the most widely used instruments to measure psychological stress. Participants rated their stress in the past two weeks on ten items (e.g., “In the past two weeks, have you felt nervous and “stressed”?”) using a five-point scale ranging from 0 “never” to 4 “all the time”. Four positively worded items were recoded, and a mean stress score was computed for each time point, with higher values indicating more perceived stress. MacDonald’s Omega was high across all measurement occasions (Ω_T4_ = .80, Ω_T8_ = .82, Ω_T11_ = .82).

#### Covariates.

Sociodemographic information was recorded at the first timepoint. We asked participants about their age (chronological), gender (male, female, other/prefer not to say), and education (eight levels, from primary education to PhD). Only 6 participants indicated the “other” option for gender and were excluded from further analyses as sample size was too small to be empirically included as a separate subgroup. Country of residence was assessed at all time points. We used the country of residence information obtained at T4 and computed four country dummy variables to be included as control variables for residency (Sweden was specified as reference country). Physical health was measured at T4 with a list of eight self-reported health conditions (High blood pressure, diabetes, heart disease, lung disease, cancer, another clinically diagnosed chronic physical health condition, a disability that affects my ability to leave the house, any other disability). Participants indicated whether they were diagnosed with any of the presented conditions since the last measurement occasion (three months ago), and we computed a sum score. Depressive symptoms in the past two weeks were assessed with the Brief Patient Health Questionnaire (PHQ-9; 9 items, answer format from 0 =  never to 3 =  nearly every day; [73]). The PHQ-9 was assessed at all measurement occasions, and we included the mean score at T4 (Ω = .93) as a time-invariant covariate.

### Analyses

**Fig 1:**
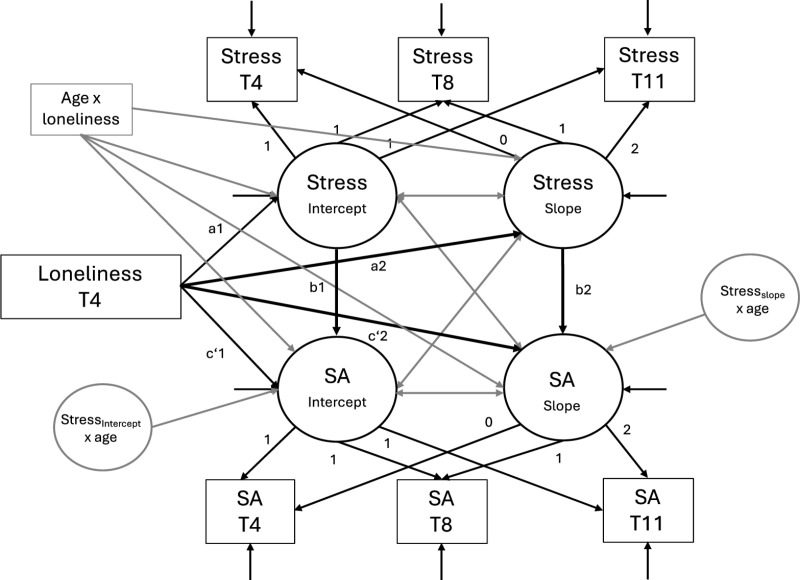
Parallel process latent growth model. **Black lines represent the effects estimated in all three models, grey lines represent interaction effects estimated only in Model 3. Covariates were regressed on all intercepts and slopes and correlated with loneliness in Models 2 and 3, however, are not presented here for reasons of clarity.** SA =  subjective age.

We first computed descriptive statistics and bivariate correlations between all variables at all time points. To model levels of subjective age and stress as well as their changes over time, we first fit two univariate latent growth models, one for each variable. To test the assumed cross-sectional and longitudinal relationships between variables and indirect effects, we computed parallel process growth models ([74]; Fig 1). In these models, loneliness at T4 was used as a predictor for both intercepts and linear slopes of stress and subjective age. Furthermore, the intercept of stress was used as a predictor for both the intercepts and slopes of subjective age, and the slope of stress predicted the slope of subjective age. We tested the indirect effect of loneliness on the level of subjective age via the level of stress (indirect effect 1, cross-sectional) and the indirect effect of loneliness on the slope of subjective age via the slope of stress (indirect effect 2, longitudinal). In the next step, we included the covariates to investigate the robustness of effects. Finally, to test whether the relations between loneliness, stress and subjective age are moderated by chronological age, we computed the interactions between age and loneliness as well as age and stress (age*loneliness, age*stress intercept, age*stress slope) and added them as additional predictors for the intercept and slope of subjective age. For the interaction of loneliness with chronological age, both variables were z-standardized, and a product term of both z-standardized variables was regressed on the intercepts and slopes of both stress and subjective age. For the interaction of stress with chronological age, we computed a latent interaction term for both the intercept and the slope of stress with chronological age (standardized), using the XWITH command [75] for interactions including latent variables in Mplus, and regressed this on the level and change of subjective age, respectively (Fig 1). For significant interactions, simple slopes analyses were performed with a macro by Preacher and colleagues [76]. Data preparation as well as descriptive analyses and bivariate correlations were computed using SPSS 29, all other analyses were computed with Mplus 8 [77]. Statistical code to reproduce the presented analyses as well as analyses outputs are available via OSF https://osf.io/76ygu/?view_only=12d6d782434c4496a36a80e149b8511a

## Results

### Descriptive statistics and preliminary analyses

Descriptive statistics and bivariate correlations for all study variables are presented in Table 1. Rank-order stabilities for stress and subjective age were high, ranging from *r* = .51 to *r* = .72. Stress and subjective age were positively correlated at all time points in a small to medium effect size range (*r* = .22 to *r* = .56), with more stress being related to an older subjective age. Higher loneliness was related to both more stress (*r* = .55 to *r* = .65) and an older subjective age (*r* = .25 to *r* = .26).

Results of the univariate latent growth models are presented in Table 2. Participants felt on average 5% younger than their chronological age (*M*_*Intercept*_ =  -.05, *p* < .001), and a significant mean slope showed that subjective age increased over time (*M*_*Slope*_ = .02, *p* < .001). There was significant interindividual variability for the intercept (σ_i_^2^ =  0.05, *p* < .001); however, there was no significant interindividual variability for the slope (σ_s_^2^ =  0.003, *p* = .138) of subjective age. As variances are not normally distributed, the Wald test might not be an optimal test for the significance of a random slope effect. We therefore compared a model with the random slope effect constrained to zero with a model where the random slope was not constrained and freely estimated. Comparing both models resulted in a nonsignificant difference (Δχ2 (2) =  3.141, *p* = .208), confirming the finding from the Wald test that there is no significant interindividual variation in the subjective age trajectories.

**Table 2:** Unstandardized regression coefficients of the univariate Latent Growth Models for Subjective Age and Stress.

Effects	Stress		Subjective Age	
	b (SE)	95% CI	b (SE)	95% CI
**Mean**				
Intercept	**1.53 (.01)**	**[1.51, 1.55]**	**–.05 (.004)**	**[–.06, –.04]**
Slope	**–.05**	**[–.06, –.03]**	**.02 (.003)**	**[.01,.03]**
**Variance**				
Intercept	**0.41(.019)**	**[.37,.45]**	**.05 (.004)**	**[.04,.06]**
Slope	**.04 (.01)**	**[.02,.07]**	.003 (.002)	[–.001,.01]
Intercept-slope covariance	**–.04 (.014)**	**[–.07, –.01]**	–.005 (.003)	[–.01,.001]
**Model fit**				
X2(df), p	0.015(1)	.90	1.73 (1)	.189
RMSEA [90% CI]	>.001	[.00,.015]	.01	[.000,.039]
CFI	1.00		1.00	
SRMR	.002		.01	

Note. Coefficients significant at *p* < .05 are printed in bold. CI =  Confidence Interval; RMSEA =  root mean square error of approximation; CFI =  comparative fit index; SRMR =  Standardized Root Mean Square Residual.

For stress, we also found a significant intercept (*M*_*Intercept*_ =  1.63, p < .001) and a significant increase in stress over time (*M*_*Slope*_ = .04, *p* < .01). Both the interindividual variances of the intercept (σ_i_^2^ =  0.24; *p* < .001) and slope (σ_s_^2^ =  0.04, *p* < .001) of stress were significant, indicating that levels and changes in stress varied considerably across individuals.

### The association of loneliness with stress and subjective age

Results of the latent growth models are presented in Table 3. In terms of cross-sectional relations, the direct effect of loneliness on subjective age (c’1) was not significant (*b* = .03, *p* = .09). Loneliness, however, was significantly and positively related to the intercept of stress (a1, *b* = .71, *p* < .001), indicating that those who were lonelier perceived more stress, and the intercept of stress was positively related to the intercept of subjective age (b1, *b* = .12, *p* < .001), suggesting that those who perceived more stress felt older. The indirect effect of loneliness on the intercept of subjective age via the intercept of stress was significant (b = .08, p < .001), which is in line with a model assuming that stress mediates the impact of higher loneliness on an older subjective age.

**Table 3:** Unstandardized regression coefficients of the multivariate Latent Growth Models without covariates (Model 1), with covariates (Model 2) and with covariates and interactions with chronological age (Model 3).

Effects	Model 1		Model 2		Model 3	
	b (SE)	95% CI	b (SE)	95% CI	b (SE)	95% CI
**Cross-sectional associations**						
Loneliness ◊ Stress intercept (a1)	**.71 (.01)**	**[.69,.73]**	**.26 (.008)**	**[.25,.28]**	**.28 (.008)**	**[.26,.29]**
Loneliness ◊ SA intercept (c‘1)	.03 (.02)	[–.01,.06]	**.02 (.009)**	**[.004,.04]**	**–.11 (.01)**	**[–.12, –.09]**
Stress intercept ◊ SA intercept (b1)	**.12 (.022)**	**[.08,.16]**	–.01 (.03)	[–.07,.05]	**.48 (.03)**	**[.42,.53]**
age ◊ SA intercept			**–.09 (.005)**	**[–.10, –.08]**	**.37 (.02)**	**[.34,.41]**
age x loneliness ◊ SA intercept					**.07 (.006)**	**[.06,.08]**
age x stress intercept ◊ SA intercept					**–.27 (.00)**	**[–.29, –.25]**
education ◊ SA intercept			**–.005 (.002)**	**[–.01, –.001]**	.002 (.002)	[–.002,.01]
gender ◊ SA intercept			–.01 (.007)	[–.02,.004]	**–.03 (.007)**	**[–.04, –.02]**
PHQ ◊ SA intercept			**.08 (.01)**	**[.05,.11]**	**–.11 (.01)**	**[–.13, –.09]**
PHC ◊ SA intercept			**.03 (.005)**	**[.02,.04]**	**.02 (.005)**	**[.01,.03]**
dummy1 (FR) ◊ SA intercept			–.005 (.01)	[–.03,.02]	–.003 (.01)	[–.02,.03]
dummy2 (DE) ◊ SA intercept			.01 (.01)	[–.01,.03]	–.005 (.01)	[–.03,.02]
dummy3 (IT) ◊ SA intercept			–.02 (.01)	[–.04,.01]	–.02 (.01)	[–.04,.003]
dummy4 (SP) ◊ SA intercept			–.02 (.01)	[–.04,.004]	**–.03 (.01)**	**[–.05, –.004]**
age ◊ Stress intercept			**–.08 (.008)**	**[–.10, –.07]**	**–.10 (.007)**	**[–.11, –.08]**
age x loneliness ◊ Stress intercept					**.04 (.006)**	**[.03,.06]**
education ◊ Stress intercept			**–.01 (.004)**	**[–.02, –.003]**	**–.01 (.004)**	**[–.02, –.004]**
gender ◊ Stress intercept			**.06 (.01)**	**[.03,.09]**	**.05 (.01)**	**[.02,.07]**
PHQ ◊ Stress intercept			**.42 (.01)**	**[.39,.44]**	**.38 (01)**	**[.35,.40]**
PHC ◊ Stress intercept			.003 (.01)	[–.02,.02]	**.02 (.009)**	**[.01,.04]**
dummy1 (FR) ◊ Stress intercept			–.03 (.02)	[–.05,.04]	.008 (.02)	[–.03,.05]
dummy2 (DE) ◊ Stress intercept			.04 (.02)	[–.01,.08]	**.06 (.02)**	**[.02,.10]**
dummy3 (IT) ◊ Stress intercept			.003 (.02)	[–.04,.05]	.02 (.02)	[–.02,.06]
dummy4 (SP) ◊ Stress intercept			**.07 (.02)**	**[.03,.11]**	**.06 (.02)**	**[.02,.10]**
**Prospective associations**						
Loneliness ◊ Stress Slope (a2)	**–.09 (.011)**	**[–.11, –.01]**	**–.05(.01)**	**[–.07, –.03]**	**–.05 (.008)**	**[–.07, –.04]**
Loneliness ◊ SA Slope (c’2)	.01 (.01)	[–.01,.02]	.00 (.005)	[–.01,.01]	**.07 (.06)**	**[.04,.11]**
Stress Intercept ↔ Stress Slope	**–.025 (.01)**	**[–.05, –01]**	**–.02 (.01)**	**[–.04, –.004]**	**–.01 (.001)**	**[–.02, –.01]**
SA Intercept ↔ SA Slope	.001 (.003)	[–.004,.01]	–.01 (.002)	[–.01,.003]	n/a	n/a
Stress Intercept ↔ SA Slope	**–.03 (.01)**	**[–.01,.004]**	.002 (.002)	[–.003,.01]	n/a	n/a
SA Intercept ↔ Stress Slope	–.002 (.004)	[–.01,.01]	.00 (.003)	[–.01,.01]	–.002 (.001)	[–.004,.00]
age ◊ SA slope			.00 (.005)	[–.01,.01]	**–.04 (.01)**	**[–.06, –.01]**
age x loneliness ◊ SA slope			–.002 (.004)		**–.05 (.01)**	**[–.07, –.02]**
education ◊ SA slope				[–.01,.002]	.00 (.002)	[–.003,.01]
gender ◊ SA slope			.002 (.006)	[–.01,.01]	–.01 (.007)	[–.02,.01]
PHQ ◊ SA slope			.005 (.007)	[–.01,.02]	.02 (.009)	[–.001,.03]
PHC ◊ SA slope			–.005 (.005)	[–.01,.01]	–.005 (.007)	[–.01,.003]
dummy1 (FR) ◊ SA slope			–.02 (.01)	[–.04,.003]	.02 (.01)	[–.01,.04]
dummy2 (DE) ◊ SA slope			–.003 (.01)	[–.02,.02]	.01 (.01)	[–.01,.03]
dummy3 (IT) ◊ SA slope			.002 (.01)	[–.02,.02]	–.003 (.01)	[–.03,.02]
dummy4 (SP) ◊ SA slope			–.009 (.01)	[–.03,.01]	.000 (.01)	[–.02,.02]
age ◊ Stress slope			**–.02 (.009)**	**[–.04, –.001]**	**–.01 (.004)**	**[–.02, –.003]**
age x loneliness ◊ Stress slope					**–.007 (.002)**	**[–.01, –.001]**
education ◊ Stress slope			–.004 (.004)	[–.01,.004]	–.002 (.001)	[–.01,.002]
gender ◊ Stress slope			–.008 (.02)	[–.04,.02]	.003 (.006)	[–.01,.02]
PHQ ◊ Stress slope			**–.03 (.02)**	**[–.06,.00]**	**–.02 (.006)**	**[–.03, –.003]**
PHC ◊ Stress slope			**.02 (.01)**	**[.001,.04]**	.008 (.005)	[–.002,.02]
dummy1 (FR) ◊ Stress slope			.007 (.02)	[–.04,.05]	–.01 (.01)	[–.03,.01]
dummy2 (DE) ◊ Stress slope			.06 (.02)	[.02,.11]	.002 (.005)	[–.02,.02]
dummy3 (IT) ◊ Stress slope			.06 (.02)	[.01,.10]	.009 (.01)	[–.01,.03]
dummy4 (SP) ◊ Stress slope			.01 (.02)	[–.04,.06]	–.006 (.01)	[–.03,.01]
**Parallel associations**						
Stress Slope ◊ SA Slope (b2)	**.14 (.065)**	**[.01,.27]**	.07 (.07)	[–.07,.21]	**1.56 (.23)**	**[1.11, 2.00]**
Stress slope x age ◊ SA Slope					**–1.09 (.17)**	**[–1.43, –.75]**
**Indirect effect**						
(1) Loneliness ◊ Stress Intercept ◊ SA Intercept	**.08 (.02)**	**[.05,.12]**	–.003 (.008)	[–.02,.01]		
(2) Loneliness ◊ Stress Slope ◊ SA Slope	**–.01 (.006)**	**[–.02, –.001]**	–.004 (.004)	[–.01,.004]		
**Explained variance (R**^**2**^)						
SA Intercept	**.19 (.03)**		**.35 (.03**)		**.80 (.02)**	
SA Slope	.28 (.29)		30 (.77)		n/a	
Stress Intercept	**.52 (.02)**		**.69 (.02)**		**.77 (.01)**	
Stress Slope	**.06 (.02)**		**10 (.03)**		.**26 (.07)**	
**Model fit**						
X2(df)	7.94(6)	.24	417.79 (48)	<.001		
RMSEA (90% CI)	.008	[.00,.02]	.031	[.028,.034]		
CFI	1.00		.96			
SRMR	.01		.03			

Note. Coefficients significant at p < .05 are printed in bold. To allow for model identification, the residual variance of the slope of subjective age had to be fixed to zero in Model 3. Models with latent interactions (Model 3) do not allow for the testing of indirect effects and no fit indices are given. CI =  Confidence Interval; PHQ =  Patient Health Questionnaire; PHC =  Physical health sum score: FR =  France, DE =  Germany, IT =  Italy, SP =  Spain. RMSEA =  root mean square error of approximation; CFI =  comparative fit index; SRMR =  Standardized Root Mean Square Residual.

Longitudinally, loneliness was not related to the slope of subjective age (c’2; b = .01, p = .325), but was significantly and negatively related to the slope of stress (a2), albeit in an unexpected direction: Those who were lonelier at T4, increased less in stress over time (b =  -.09, p < .001). As expected, the slope of stress was positively related to the slope of subjective age (b2; b = .14, p = .03), indicating that those whose perceived stress increased over time also felt older over time. Again, the indirect effect was significant, in line with a mediation model assuming that the effect of loneliness on the slope of subjective age was mediated by the slope of stress (b =  -.01, p = .039).

Next, we included chronological age, gender, education, depressive symptoms, physical health, and country as covariates into the models (Table 3, Model 2). This rendered many of the previous effects non-significant, most notably the effect of the slope and intercept of stress on the slope and intercept of subjective age (b-paths), which also rendered the indirect effects non-significant. The effects of loneliness on both the intercept (b = .26, p < .001) and slope (b =  -.05, p < .001) of stress remained significant; furthermore, the direct effect of loneliness on the intercept of subjective age now became significant (b = .02, p = .016), indicating that cross-sectionally, feeling lonelier is related to an older subjective age.

As for the covariates, higher depression scores were cross-sectionally related to an older subjective age (b = .08, p < .001) and more stress (b = .42, p < .001), whereas an older chronological age and higher education were related to a relatively younger subjective age (b_age_ =  -.09, p < .001; b_edu_ =  -.01, p = .025) and less stress (b_age_ =  -.08, p < .001; b_edu_ =  -.01, p = .007). More physical health problems were related to an older subjective age (b = .03, p < .001), and women perceived more stress than men (b = .06, p < .001). There were no significant relations of the covariates on the slope of subjective age, whereas an older chronological age (b =  -.02, p = .041), and more depressive symptoms (b =  -.03, p = .047) predicted less increase in stress, and more health problems were related to a higher increase in stress (b = .02, p = .037). Though there were no consistent effects of the country dummies on the relation between loneliness, stress and subjective age, we repeated all analyses for each country and present these findings in the OSF (https://osf.io/76ygu/?view_only=12d6d782434c4496a36a80e149b8511a). While there were some differences in the effects, the overall pattern of results was consistent. Due to the small sample sizes at T11, which led to estimation problems in some models, and the fact that models 2 and 3 did not include the country dummies as covariates, these models need to be interpreted with caution.

### The moderating role of chronological age

**Fig 2:**
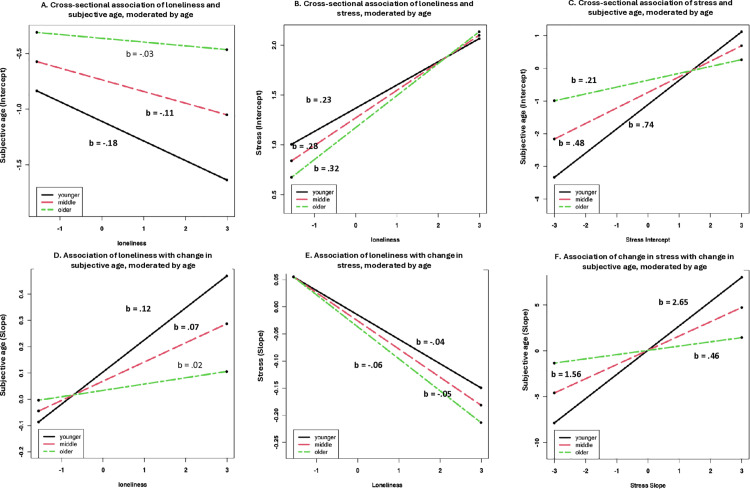
Graphical depiction of the interaction effects and simple slopes from the latent growth model (Model 3). Intercepts represent the initial estimated scores; slopes represent the estimates of linear change. Younger =  younger adults (1 SD below the mean, 34.42 years), middle aged =  middle aged adults (Mean, 50.41 years); older =  older adults (1 SD above the mean, 66.4 years). Coefficients in bold print are significant at p < .05.

In the final model, we included age interactions on all paths from loneliness to stress and subjective age, and from stress to subjective age, respectively (Table 3, Model 3). All interaction effects reached statistical significance, albeit in different directions (Fig 1). For the cross-sectional model, the effect of loneliness on the intercept of subjective age turned negative. Given that both the bivariate correlation and the effect in previous models were positive, this might be a case of statistical suppression [78]. This effect was qualified by a positive age moderation. Simple slope analyses (Fig 2, Panel A) indicated that higher loneliness was related to a younger subjective age, but only for younger (b =  -.18, p < .001), and middle-aged (b =  -.11, p = .001) participants, whereas there was no significant relationship for older participants (b =  -.03, p = .29). The relation of loneliness with the intercept of stress (Fig 2, Panel B) was most pronounced for older adults (b = .32, p < .001), indicating that less loneliness was related to less stress especially for older participants. In terms of the relationship of stress with subjective age (Fig 2, panel C), this was the other way around, as the association of stress with subjective age was most pronounced for younger adults, for whom less stress was most strongly related to a younger subjective age (b = .74, p < .001).

In terms of longitudinal relations, higher loneliness was related to a larger increase in subjective age over time but only for younger (b = .12, p < .001) and middle-aged participants (b = .07, p < .001; see Fig 2D). Furthermore, the effect that greater loneliness was related to a larger decrease in stress was especially pronounced for older adults (b = .06, p < .001; see Fig 2E). Finally, more stress was related to a larger increase in subjective age, especially for younger adults (b =  2.65, p < .001; see Fig 2F).

## Discussion

The aim of the current study was to investigate the association between loneliness and subjective age, a well-proven indicator for and predictor of adaptive development. We go beyond previous studies by investigating stress as one potential pathway through which loneliness and subjective age are related in a sample covering the entire adult life span. We further aimed to uncover whether associations between loneliness, stress and subjective age vary across adulthood. In a longitudinal study spanning 2.5 years, a large group of participants from five different countries reported on their loneliness, perceived stress, and subjective age. Cross-sectional findings show that more loneliness was related to an older subjective age via higher stress. Longitudinally, there was also an indirect effect of loneliness on subjective age via stress, although contrary to our hypotheses in that higher loneliness was related with decreased in stress over time, and consequently, subjective age. Longitudinal effects mostly vanished when including several sociodemographic and health-related covariates, as did the relationship between stress and subjective age, while loneliness and subjective age remained significantly interrelated. All associations between the three variables were moderated by chronological age, even when covariates were included, highlighting the importance of a lifespan approach when investigating factors associated with subjective age and its trajectories.

### Loneliness is related to stress and subjective age – but not uniformly

Even though there was no direct effect of loneliness on the level or slope of subjective age, cross-sectional results show that participants who reported more loneliness also felt more stressed and reported an older subjective age. This is in line with previous findings showing that the experience of loneliness is a major stressor (e.g., [14]) and supports previous studies cross-sectionally linking loneliness to aversive aging experiences (e.g., [25,50]). As these findings are cross-sectional, however, there might be potential third variables causing the associations, for instance reductions in physical and social activities at the beginning of the pandemic.

Longitudinally, the slope of stress predicted the slope of subjective age in the assumed direction, those who increased more in stress also felt older over time. This adds to evidence from previous studies that identified stress as one major predictor of subjective age in different populations over different time frames [1,11,12,79].

Contrary to our expectations, however, participants who were lonelier at T4 reported smaller increases in stress, and in turn, smaller increases in subjective age. As we did not include loneliness at more time points, one explanation might be that some people might have recovered from their loneliness after the pandemic (e.g., when contact restrictions were lifted or with increase in social support), especially those who felt very lonely. This could then also lead to a decrease in stress for those participants, which might not have been so pronounced for those who weren’t that lonely in the first place. Indeed, some studies reported that the impact of the pandemic onset on loneliness and satisfaction with social contacts was temporary and that processes of recovery and psychosocial adaptation unfolded with increasing duration of the pandemic [15,17,80]). Our results might also relate to findings reported in the systematic review by Brown and colleagues [13], who highlighted that several studies found that loneliness was related to decreased stress responses for some physiological outcomes (e.g., heart rate), which they interpreted as a blunted stress response, also detrimental to health [13]. Future studies should replicate these findings and link physiological and self-report indicators of stress. Another explanation could be regression toward the mean or a ceiling effect, as lonelier individuals had already high stress levels at baseline so that their stress scores could hardly increase any further over time.

Many of the relationships, in particular those between stress and subjective age, were less consistent, and reduced or even vanished when including age, education, gender, depressive symptoms and health problems as covariates. This might be an indication for potential mediating mechanisms, e.g., of depressive symptoms or health problems, in the relationship between stress and subjective age. Given that both self-reported and physiological indicators of stress have been linked with subjective age in prior research [11, 12]), identifying the psychological and physiological mechanisms of this relation could be the next step to address and counteract the influence of stressful experiences on the aging process.

Notably, the relation between loneliness and stress remained robust, and in the model with covariates, loneliness was also positively related to the intercept of subjective age, indicating older subjective ages of individuals reporting higher levels of loneliness. When controlling for chronological age, education, gender, country and physical as well as health problems, the experience of loneliness as a pervasive stereotypic feature of late life [48] might be seen as a marker of getting older and thus, trigger an older subjective age. Other variables, such as personality traits [81] or health [82] have also been identified as predictors of subjective age, so future studies should investigate the relative and unique role of loneliness in a larger nomological network of variables influencing subjective age (e.g., [83]). In addition, it is very likely that stress is not the only mediator of the association between loneliness and subjective age. Other behavioral mediators, such as health behaviors and social activities which are influenced by loneliness might consequently affect subjective age (e.g., [71]). Following assumptions by both SST and SAVI, more general loneliness-induced negative affect might also be relevant for subjective age, above and beyond perceived stress. And finally, physiological indicators, such as loneliness-induced heightened cardiovascular response, should also be addressed as potential mediators.

As we did not model loneliness as a time-invariant predictor, we cannot rule out reverse causation, in that feeling older causes stress and reduces motivation to engage in certain activities and social gatherings ([84–86]), thereby increasing loneliness. This needs to be tested in future studies. This is a general problem of mediation analyses, particularly in a cross-sectional context, that various alternative causal mediation models might result in a model fit that is as good as the targeted mediation model [87]. Nevertheless, as loneliness was also systematically related with changes in stress and subjective age, at least these temporal associations are unlikely to be a result of reversed causation.

Furthermore, the random slope component of subjective age was not significant, limiting our ability to detect longitudinal mediation effects. Still, although the slope variance was not significantly different from zero, several associations of subjective age slopes with other variables were statistically significant, and the relations between these three variables show that it might be worthwhile to tackle any of them with interventions, and thus, also have positive effects on the other two. These findings should still be interpreted with caution, as they might not represent a robust relationship. Future studies should reconsider these relationships and test them with more dynamic measures of loneliness and in more detail model the influence of historical events that might have led to especially high loneliness levels in particular times. We will also discuss potentially different, nonlinear trajectories of subjective age in the next section.

### The moderating role of chronological age on associations between loneliness and stress

All reported associations were qualified by significant interactions of both loneliness and stress with chronological age. Given the large age range of our sample, this was expected, as the predictors of subjective age and its relevance might be different depending on life stage [88–91]. Furthermore, theoretical frameworks, such as SAVI and SST also assume differential impacts and implications of both social isolation and stress across life [58,62]. Contrary to our expectations, loneliness seemed not to affect the level or change in subjective age of older participants, neither in the positive, nor negative direction. Regarding their subjective age, older participants might thus be less inclined to integrate the experience of loneliness into their age identity. It is also possible that loneliness is more expected in later life, and thus, does not additionally contribute to an older subjective age while it is less expected in younger and middle-aged adults, and might thus be regarded by them an important marker of “getting older”. In terms of stress, when loneliness was low, stress was also lowest for older adults, however, there were no age differences in stress at high levels of loneliness. This is at first sight somewhat in contrast to expectations of both SST and SAVI, which would assume that loneliness has a stronger impact on older adults. Thinking about the strengths of older adults, though, the experience of satisfying social relationships might be especially helpful to boost the well-being of older adults, as older adults tend to prioritize emotional and social goals [58]. Furthermore, older adults might be particularly adept to leverage their social relations to buffer stress. In terms of the lack of age differences at high levels of loneliness, this might be due to the specific situation in the pandemic, where loneliness was a somewhat collective experience across all age groups [92] and might thus not have been perceived as an enduring, stressful situation.

Results were more consistent with our expectations regarding the relationship of stress with subjective age. Here, our findings show that elevated stress seems to be most relevant for the subjective age of younger adults, both in terms of levels as well as changes [93]. This is in line with previous findings by Wettstein and colleagues [12] who found that the association of stress with subsequent subjective age was weaker for older adults. In later life, subjective age might be affected more by other factors, such as health problems, or the (downward) comparison to same aged peers, and less on stress experiences, while for younger and middle-aged adults, stress might trigger age-associated interpretative frameworks for experiences and physiological processes that lead to an older felt age [94]. Moreover, stressor types vary across the lifespan, with older adults typically no longer affected by work-related stressors [61]. There also seems to be an age-related increase in the use of adaptive coping strategies that mitigate the impact of stressors [27]– at least until early-old age [95]. This could be another explanation for weaker stress-subjective age interrelations in chronologically older individuals. The timing of our study in the pandemic context might also play a role, as several studies have found that older adults seemed to have experienced less stress and showed more resilience during the pandemic compared to younger adults. (e.g., [26,63]. Further studies should look more in detail into the differential relationship of different stress indicators, specific stressors, hassles as well as stress processes and stress reactions with the experience of aging in different age groups and in different historical contexts.

### Limitations

Our study has several strengths, such as the large and representative sample of participants in a broad age range covering the entire adult lifespan, and from different countries. Furthermore, our study design allowed us to investigate cross-sectional as well as longitudinal relationships during the Covid-19 pandemic and beyond. Nevertheless, we also note several limitations that might impact the interpretation of our results and should be addressed in future research.

We had a large dropout at T11, which considerably reduced sample size at the third measurement occasion for the current analyses and did also not allow us to model loneliness as a dynamic, time-varying variable, due to low covariance coverage. Furthermore, the dropout reduced the representativeness of the sample, which was high at earlier measurement occasions. Nevertheless, our selective attrition analyses revealed that differences between the subsamples of dropouts and non-dropouts were all in a small effect size range, and we used Full Information Likelihood estimation and included dropout-informative covariates (such as depressive symptoms) to minimize estimation bias due to selective dropout.

Another limitation related to our sample’s representativeness that we excluded participants who did not identify as either male or female due to the extremely small number of participants in this category (*n* =  6). As queer and gender diverse participants are often underrepresented in aging research, future studies should aim at increasing the sample sizes of more diverse gender identities to ensure inclusivity and increase in knowledge on aging beyond binary gender conceptions.

In addition, as subjective age was only assessed three times, our models were restricted to the estimation of linear effects, while there might be more complex, nonlienar subjective trajectories to be uncovered [2,21,96]). This might also be one explanation for the non-significant slope variability in subjective age, as our three-wave design only allowed to test for linear change, which might not represent actual subjective age changes, including context- and pandemic-related “ups and downs”, over time. As a first indication of this, we computed the intraclass correlation for subjective age and found that while 58% of variability was between persons, a substantive amount of variability (42%) was within person. Thus, there seems to be intraindividual variation in subjective age, which is just not well represented by a linear trend.

Relatedly, we did not include time-varying predictors to avoid overcomplex models. Constructs such as depressive symptoms may change over time, and their change might have an impact on the reported relations. In general, most of the obtained effects were rather small in size, so future studies with a priori established, sufficient statistical power and more measurement occasions are needed to further evaluate the robustness of our findings.

In terms of timing, our study started during the Covid-19 pandemic, which might not be representative for previous times regarding our study variables. To test these effects, however, a pre-pandemic measurement occasion would have been required to distinguish levels before the pandemic from the dynamics that unfolded during and after the pandemic. In a similar vein, as the measurement of subjective age only took place in November 2021, February 2022, and June 2023, two of our three measurement occasions were at the end or even after the pandemic, so that we neither can make any claims about relationships in the midst of the pandemic, nor can we investigate how variable relations developed during this time and might thus have affected our results.

Another limitation in terms of study design is that the time frame of investigation was rather short, with three measurement occasions spanning two and a half years. Since both loneliness and stress seem to be especially detrimental when they become chronic ([59,97], longer time frames need to be investigated. Additionally, and given that the slope for subjective age was not significant, a longer observation period based on more than three measurement occasions might have resulted in more interindividual variation in subjective age trajectories and, consequently, in stronger associations of loneliness and stress with these trajectories. For the investigation of processes linking loneliness to momentary stress and subjective age, however, shorter timeframes, such as ecological momentary designs might be helpful to uncover how these variables are linked in participants daily life [11]. In sum, the integration of different timelines might be optimal for future studies to better understand how short-term mechanisms translate into long-term relationships and vice versa [97].

In terms of measures, stress was only assessed via a general measure of self-report. While this is an important operationalization of stress [64,66]) that is systematically related with subjective age [1,12];, including physiological measures of stress, such as salivary cortisol or cardiovascular measures ([11]) might be important to investigate whether loneliness and stress affect subjective age also via bodily mechanisms and their interpretation (e.g., [98, 99] and whether physiological or subjective stress is more strongly related with loneliness and subjective age. Furthermore, more concretely asking about the type of stressor might shed light on the mechanisms behind age differences

Finally, while we include a sample of participants from five different countries and all our main constructs (subjective age, stress, loneliness) were operationalized by instruments or scales widely used and validated in different cultures (e.g., [2,100,101]), we did not systematically investigate country differences in the assumed relations, as this goes beyond the scope of the present analyses. This should be addressed in future studies, as culture plays an important role for the subjective experience of aging [102].

## Conclusion

Our findings shed light on the cross-sectional and longitudinal relations between loneliness, stress, and subjective age. We show that the experience of loneliness, which is a stereotypical, negative feature of old age is indeed negatively related to individual aging experiences, and that stress might be one relevant mediating factor contributing to this relation. Moreover, associations between loneliness, stress and subjective age vary according to chronological age. This adds to the large body of evidence regarding the importance to combat loneliness on individual and societal levels – also for the sake of maintaining positive subjective aging experiences and its beneficial consequences for health and well-being - and further highlights the importance of lifespan considerations while doing so.
